# Treadmill locomotion in the American alligator (*Alligator mississippiensis*) produces dynamic changes in intracranial cerebrospinal fluid pressure

**DOI:** 10.1038/s41598-022-15918-9

**Published:** 2022-07-12

**Authors:** Bruce A. Young, Michael J. Cramberg

**Affiliations:** Department of Anatomy, Kirksville College of Osteopathic Medicine, A.T. Still University, Kirksville, MO 63501 USA

**Keywords:** Neuroscience, Medical research

## Abstract

To examine the influence of movement on cerebrospinal fluid (CSF) dynamics, intracranial subdural pressure recordings were taken from sub-adult alligators (*Alligator mississippiensis*) locomoting on a treadmill. Pressure recordings documenting the cardiac, ventilatory, and barostatic influences on the CSF were in good agreement with previous studies. During locomotion the CSF exhibits sinusoidal patterns of pressure change that spanned a mean amplitude of 56 mm Hg, some 16 × the amplitude of the cardiac-linked pulsations. These sinusoidal CSF pulsations were closely linked to the locomotor kinematics, particularly the lateral oscillations of the alligator’s head. Data recorded from the freely moving alligators suggest that fluid inertia, body cavity pressures, and likely other factors all influence the CSF pressure. The clear relationship between movement and CSF pressure described in this study suggests that the paucity of studies examining human CSF dynamics during movement should be addressed.

## Introduction

The cerebrospinal fluid (CSF) plays a key role in the development^1^ and metabolic maintenance^2^ of the central nervous system. In order to fulfill its diverse functional roles, the CSF must circulate; studies have postulated that abnormal CSF circulation may be the causal agent for a variety of neurological disorders, including Alzheimer's^3^ and syringomyelia^4^. Despite the importance of the CSF fluid dynamics, little is known about the magnitude, or even pattern, of "normal" CSF flow^5^. The CSF exhibits pressure pulsations corresponding to the cardiac cycle^6^; these pulsations have been thought to influence the dynamics of the CSF within the skull^7^, but this interpretation has been challenged^8^. Studies have shown that the ventilatory cycle generates significant levels of CSF flow^9^, primarily along the spinal cord^10^. Changes in the internal pressure of the body cavities, the intrathoracic and intraperitoneal pressures, can also influence the CSF dynamics^11^; it is unclear how these cavity pressure changes relate to the cardiac and ventilatory influences^12^.

The emphasis on cardiac and ventilatory forces acting on the CSF reflects the underlying experimental evidence. Almost all of the data on CSF fluid dynamics were collected surgically on anesthetized animal or human subjects, or were recorded from human or animal subjects using magnetic resonance imaging (MRI)^13^. These different approaches for CSF data collection have two common features: 1) they provide clear records of cardiac and ventilatory patterns^14^, and they are largely incompatible with a moving subject^15^. Experimental stimulation of the myodural bridge, skeletal muscle fibers that insert onto the dura, demonstrated that muscle contraction can directly influence CSF pressure^16,17^. Recent work revealed that the undulatory movement of a snake's body, during fictive or conscious locomotion, resulted in CSF pressure pulsations of greater magnitude than those associated with either the cardiac or ventilatory cycles^18^.

The present study was undertaken to see if limbed locomotion has similar influence on CSF pressure as was shown with vertebral displacement in snakes. During terrestrial locomotion the American alligator (*Alligator mississippiensis*) employs a range of limb kinematics, with the main variable being the range of flexion in the limb. With high degrees of flexion at the shoulder/pelvis and elbow/knee, the proximal portion of the limb is held nearly horizontal; in this “push-up” position the ventral surface of the body is close to the substrate, so this is referred to as the “low walk.” With lower degrees of flexion at the shoulder/pelvis and elbow/knee, the proximal portion of the limb is held more vertical; in this semi-erect position the ventral surface of the body is further from the substrate, so this is referred to as the “high walk”. The low walk and high walk represent a functional continuum. Associated with this continuum is a range of lateral displacement of the limb (being greater in the low walk than the high walk) and corresponding lateral oscillation of the head, trunk, and tail base (again, being greater in the low walk than the high walk). Alligators have anatomical specializations to limit (but not eliminate) the undulatory displacement of the axial skeleton during locomotion^19^. Alligators are capable of shifting limb mechanics mid-stride and generally exhibit a wide range of limb angles^20^; because of this kinematic plasticity, the relationships among limb angle, body undulation, and locomotor velocity are complicated in *Alligator* and all crocodylians. By first training sub-adult alligators to locomote on a treadmill, it was possible to directly record intracranial CSF pressure from an unanesthetized vertebrate performing normal limb-based locomotion.

## Results

**Figure 1:**
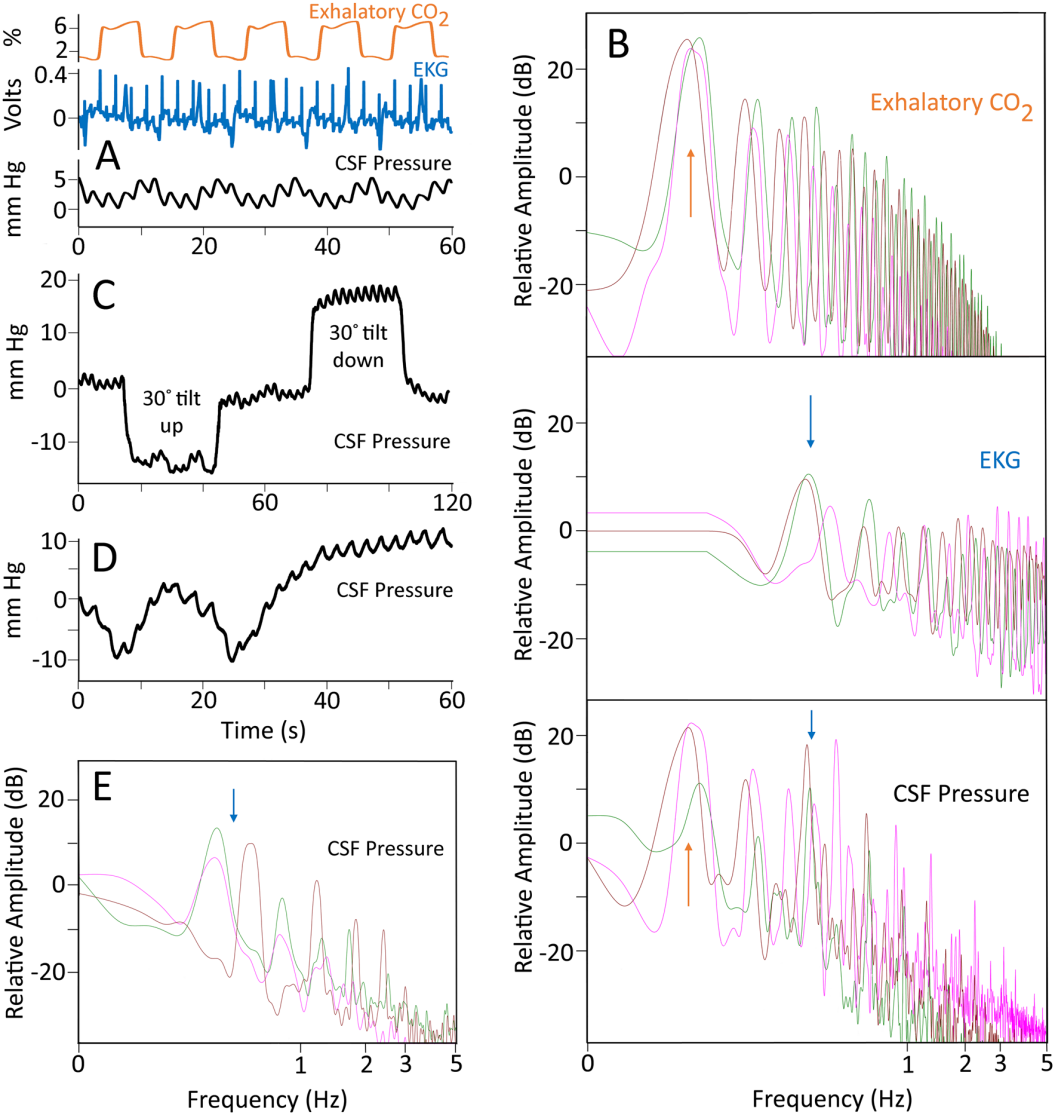
Recognition and stability of the CSF pulsations linked to the cardiac cycle. (**A**) Simultaneous data traces recorded from a 146 cm *Alligator mississippiensis* during initial anesthesia the CSF pressure pulsations show a higher-frequency pattern that corresponds to the cardiac cycle, as evident in the EKG traces, and a lower frequency pattern that corresponds to the ventilator cycle, as evident in the exhalatory CO_2_. (**B**) Power spectral analyses of similar data sets from three different alligators (color coded in figure); the exhalatory CO_2_ has a dominant frequency of 0.08 Hz (vertical arrow), the EKG has a dominant frequency near 0.4 Hz (vertical arrow), and the simultaneously recorded CSF pressure reveals frequency spikes in both regions. (**C**) The CSF response to orthostatic gradients was used to ensure the integrity of the preparation. (**D**) After recovery from anesthesia, when the animal was still between bouts of locomotion, clear cardiac-related pulsations in the CSF were recorded. (**E**) Power spectral analyses of the CSF cardiac pulsations recorded between locomotor bouts from three alligators (color coded) reveal the same dominant frequency (vertical arrow) recovered earlier from the EKG and CSF traces. In these records, the CSF pressure is indicated in black, the EKG in blue, and the exhalatory CO_2_ in orange.

With the alligator anesthetized and on forced ventilation, there were two clear frequencies of pulsation within the intracranial CSF (Fig. 1A), a high-frequency that corresponded to the cardiac cycle (recorded as EKG) and a low frequency that corresponded to the ventilatory cycle (recorded as exhalatory CO_2_). Power spectral analysis of the exhalatory CO_2_ data revealed a dominant frequency at 0.08 Hz (Fig. 1B) corresponding to the ventilation rate of 5 breaths per minute; power spectral analysis of the EKG records revealed a dominant frequency near 0.4 Hz (Fig. 1B) corresponding to a heart rate of approximately 24 beats per minute. Power spectral analysis of the CSF pressure data recorded while the alligator was on forced ventilation reflects the combined influence of the ventilatory and cardiac cycles as both dominant frequencies are evident in the CSF spectra (Fig. 1B). During this baseline, the (anesthetized) alligators had a mean heart rate of 22 beats per minute (s.d. = 5.0) and the associated CSF pulsations had a mean amplitude of 3.7 mm Hg (s.d. 0.19).

The integrity of the experimental preparation was tested by exposing the anesthetized alligators to orthostatic gradients. Tilting the alligators 30° head-down caused an increase in CSF pressure of 18.6 mm Hg (s.d. 1.23), and a slight increase in the amplitude of the cardiac-linked CSF pulsations (Fig. 1C). Tilting the alligators 30° head-up caused a decrease in CSF pressure of 16.8 mm Hg (s.d. 2.4), and a slight decrease in the amplitude of the cardiac-linked CSF pulsations (Fig. 1C). Post-recovery from anesthesia, the alligators were placed on the treadmill. They would exhibit short periods of locomotion, then would remain stationary, either holding their body off the surface of the treadmill, or "sprawling" out onto the tread. CSF pulsations recorded during these stationary periods (Fig. 1D) had a mean frequency of 0.39 Hz (or 23.9 beats per minute, s.d. = 4.7) and mean amplitude of 3.8 mm Hg (s.d. = 0.19); these were not significantly different (for frequency: t = 0.598, *p* = 0.572, df = 6; for amplitude: t = 0.989, *p* = 0.329, df = 34) than the cardiac-linked pulsations recorded while the animal was under anesthesia. Power spectral analysis of the CSF pressures recorded when the animal was not being actively ventilated revealed simpler spectra (Fig. 1E) with dominant frequencies which matched those of the earlier EKG spectra.

**Figure 2:**
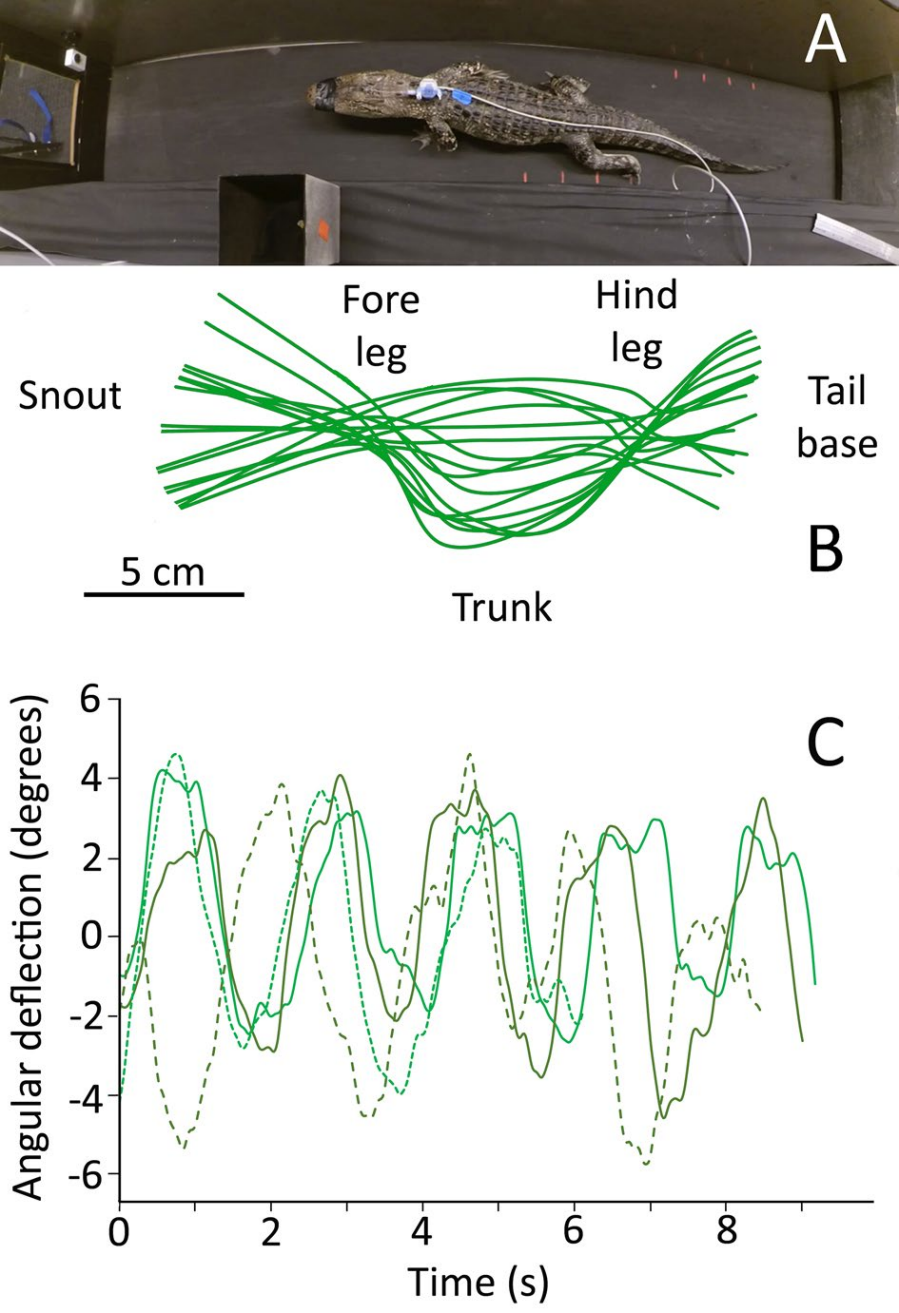
Kinematics of treadmill locomotion in *Alligator mississippiensis*. (**A**) dorsal image of a 161 cm *Alligator mississippiensis* locomoting on the treadmill after the surgical attachment of the pressure catheter and transducer. (**B**) traces along the dorsal midline of a 150 cm *Alligator mississippiensis* locomoting on the treadmill. The front and hind legs form undulatory nodes, the head oscillates in the horizontal plane during each step cycle. (**C**) sinusoidal waves of lateral head deflection recorded during locomotor bouts from four different alligators. These waves were centered to 0 for ease of presentation.

When locomoting on the treadmill the alligators used a mix of "low-walk" and "high-walk" postures (Fig. 2A). Kinematic analysis demonstrated that their footfall pattern was coupled to (low amplitude) lateral deflections of the vertebral column. Midline traces of the alligator's body demonstrated that the lateral undulations of the body were lowest over the limbs (which serve as undulatory nodes) and increased at the tip of the snout, the mid-point of the trunk, and at the tail (Fig. 2B). With each step cycle, or sequence of footfalls, the head of the alligator swung back and forth in the horizontal plane (Fig. 2B). The angular displacements of the midline of the head relative to the midline of the neck (which is more stationary due to the nodal role of the forelimbs, Fig. 2B), formed a sinusoidal series (Fig. 2C) with a mean range of 6.4 degrees (s.d. = 1.6). Video analysis found no difference in the locomotor velocity, or kinematics, between the training and post-surgical trials; since anesthesia was not used during the treadmill training, this indicates that the alligators were adequately recovered from the anesthesia when the locomotor data were collected.

**Figure 3:**
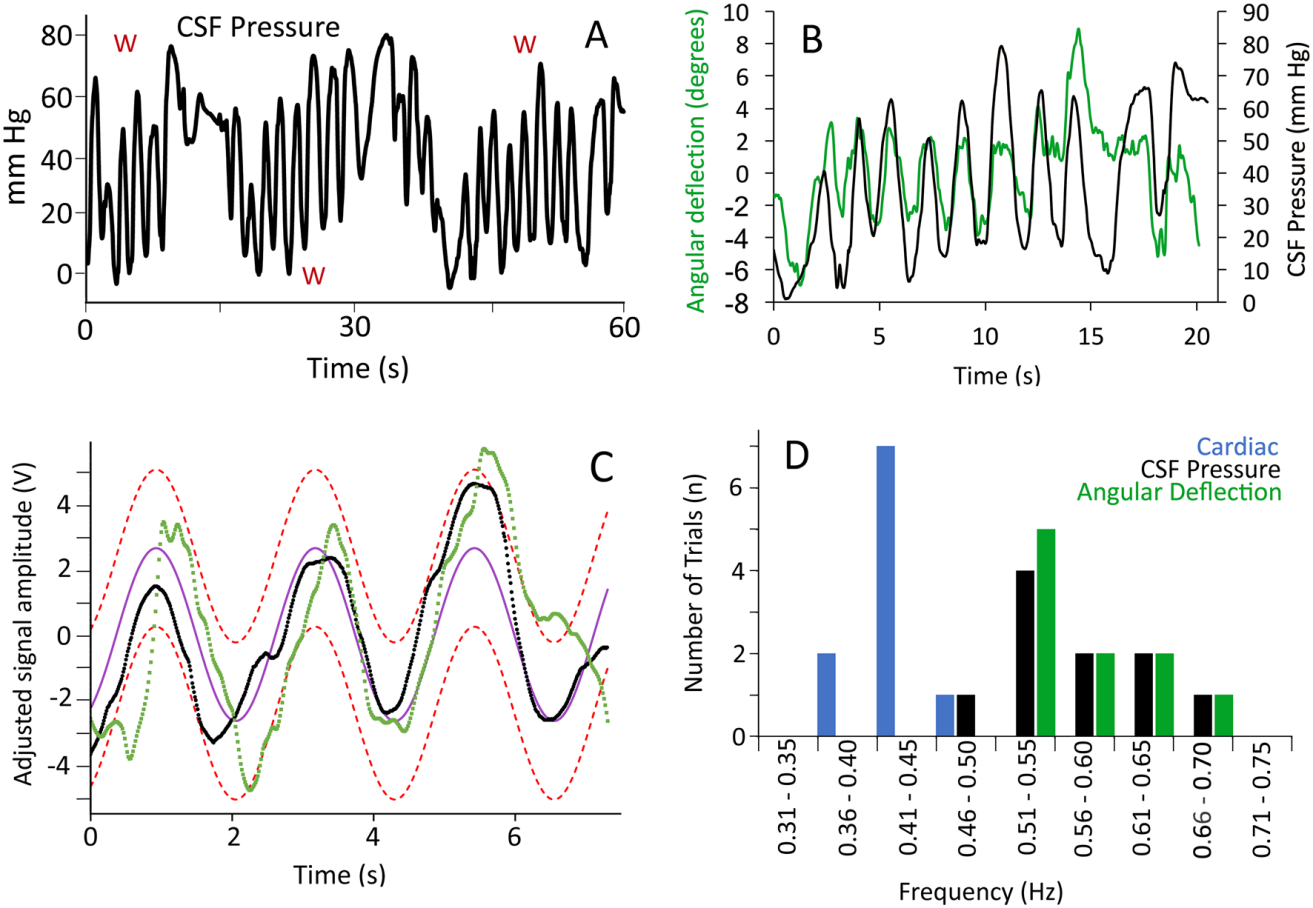
Changes in CSF pressure during locomotion. (**A**) continuous raw data trace of CSF pressure from an alligator on the moving treadmill; the periods of continuous locomotion (indicated by a red W) were broken up by the alligator making contact with the walls or end of the treadmill, or if the animal simply stopped walking. (**B**) synchronized raw data showing the temporal congruence between the lateral oscillations of the head (green) and the pulsations in the CSF pressure (black). (**C**) to explore their congruence, the CSF pressure and head oscillation curves were both centered at 0, then their amplitudes matched. A sinusoidal curve (purple line) was then optimized to fit the CSF pressure curve (back trace), and a 95% confidence interval calculated for the sinusoidal curve (red dashed lines). The majority of the head oscillation curve (green trace) fits within the 95% confidence interval. (**D**) Distribution of the dominant frequency (determined using Power spectral analysis) of the cardiac pulsations (blue), CSF pressure pulsations (black), and undulatory kinematics of the head (green); the cardiac frequencies (which are similar to those detailed earlier) are lower than the CSF and kinematic frequencies which are closely aligned.

When the animal was locomoting over the treadmill the CSF pressure underwent low frequency (mean 0.54 Hz, s.d. = 0.09) sinusoidal changes (Fig. 3A) with mean amplitudes of 59.5 mm Hg (s.d. = 12.19). These high-amplitude sinusoidal pressure cycles stopped as soon as the animal stopped moving (Fig. 3A); if the animal remained stationary the cardiac-linked CSF pulsations were again observed. The frequency of the CSF pulsations increased with increasing locomotor velocity of the alligator (tested hypothesis that the regression coefficient was = 0; F = 0.283, *p* = 0.608, df = 1, R = 0.17). In all of the locomotor trials, there was a close temporal congruence between the lateral oscillations of the alligator's head during locomotion, and the sinusoidal pattern of CSF pulsations (Fig. 3B). Regression analysis revealed a significant (F = 51.26, *p* = 0.00005, df = 1, R = 0.92) relationship between increasing oscillatory frequency of the head and the frequency of the CSF pulsations. To examine this frequency relationship further, the CSF locomotor pulsations were modeled as a sinusoidal wave, then a 95% confidence interval calculated for the model wave. The recorded CSF pressure consistently fit within the confidence interval, and there was significant overlap between the wave pattern of head oscillation and the modelled sinusoidal curves (Fig. 3C). Power spectra analysis was performed on: 1) identified cardiac-related CSF pulsations recorded during the locomotor trials, 2) CSF pressure recordings during locomotion, and 3) kinematic data on the angular deflection of the head. This analysis revealed a clear segregation between the dominant frequencies of the cardiac- and locomotor-related pulsations (Fig. 3D) and a good match between the locomotor CSF pulsations and the corresponding kinematics of the head (Fig. 3D).

**Figure 4:**
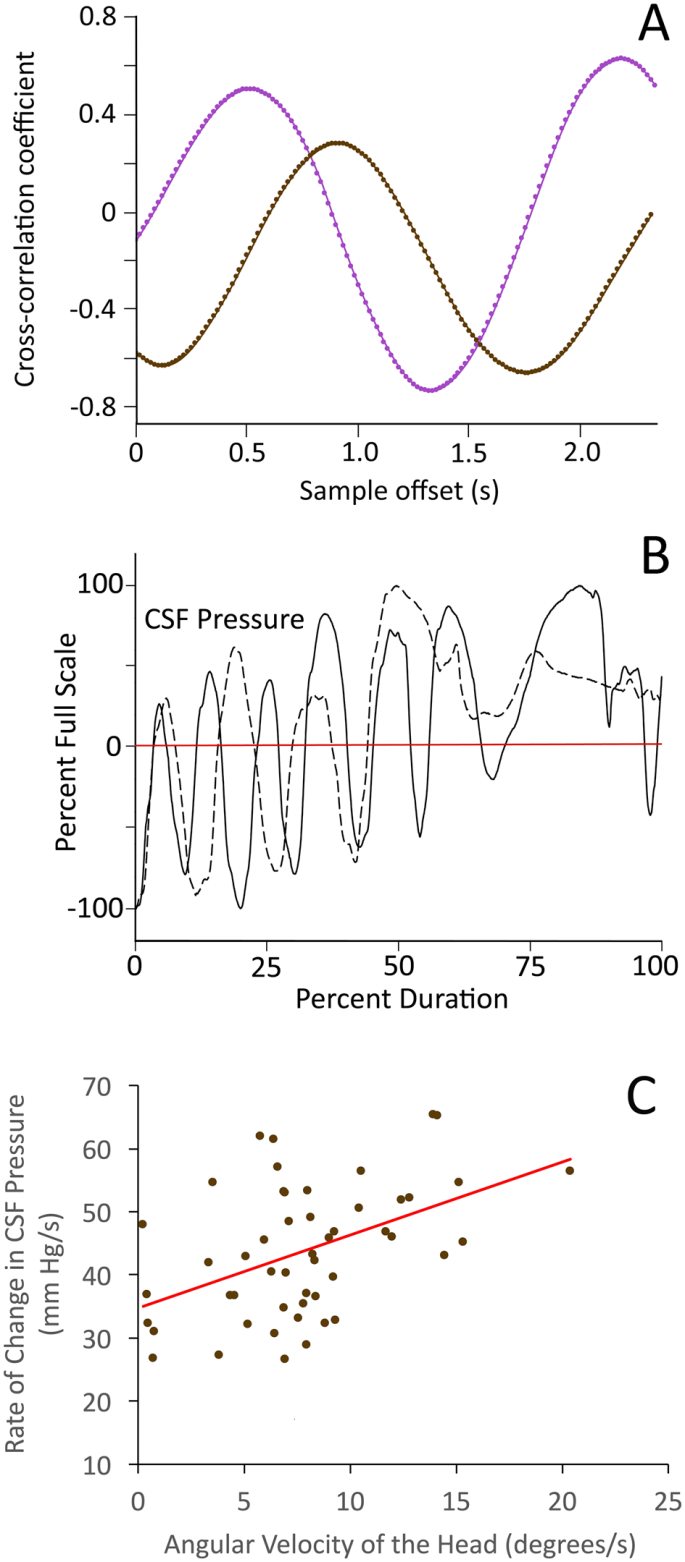
Temporal and amplitude patterns in the locomotor-based CSF pressure waves. (**A**) Cross-correlation analyses from two alligators (color coded) produced when the CSF pressure record is sequentially offset against the kinematic data on head rotation. Both cross-correlation analyses recovered a sinusoidal curve with a wavelength corresponding to those determined from the CSF and kinematic analyses, but with varying positive and negative correlation coefficients reflecting the variation in the amplitudes between the two data sets. (**B**) CSF pressure traces recorded during two locomotor sequences, plotted as percent scale. In addition to the pattern of sinusoidal curves, the traces exhibit an increase in CSF pressure during locomotion. Most of the CSF pressure curve is below the average at the onset of locomotion, but at the end of locomotion the CSF pressures are above the average. (**C**) Relationships between CSF pressure changes and locomotor head movements. Using raw (non-adjusted) data, the rates of head oscillation and CSF pressure change were compared. The CSF pressure increases significantly faster when the head is oscillated faster, but this relationship does not explain the majority of the variation (R = 0.47) because it does not include the gradual increase in CSF pressure.

The locomotor kinematics and CSF pressure had good congruence in the frequency domain (Fig. 3B–D), but less congruence in the amplitude domain (Fig. 3B–D). Cross-correlation analysis of the locomotor CSF and kinematic records produced the expected sinusoidal curve of regression coefficients (Fig. 4A), with wavelengths corresponding to the dominant frequencies determined through power spectral analysis (Fig. 3D). The determined correlation coefficients ranged widely, reflecting the variation in the amplitudes in the two data records (Fig. 4A). This amplitude variation is caused, at least in part, by the fact that there are two trends in CSF pressure evident during locomotion. In addition to the sinusoidal curves that correspond to the angular displacement of the head, there is a trend of increasing baseline CSF pressure over the course of the locomotor series (Figs. 3A, 4B). To compare the magnitude of the head oscillations and the CSF pulsations, the rate of head oscillation was compared to the rate of CSF pressure change during locomotion (Fig. 4C). There was a significant (F = 13.38, p = 0.0006, df = 1) relationship between increasing head oscillations and increasing CSF pressure (Fig. 4C) but this relationship only explains about half (R = 0.47) of the variation in CSF pressure observed during alligator locomotion, the remaining CSF pressure variation stems from the gradual elevation of pressure during movement.

## Discussion

The present study was undertaken to test the hypothesis that unrestrained limbed locomotion would result in a novel pattern of large amplitude pulsations in the cerebrospinal fluid (CSF). Sub-adult alligators, previously trained to walk on a treadmill, exhibited similar kinematic patterns before and after a fluid pressure sensor was implanted into their cranial subdural space. The CSF exhibited a clear and consistent pattern of pulsations linked to the cardiac cycle (Fig. 1A, C, D); these pulsations, the effect of tilting on the CSF pressure, and the alligator’s heart rate all agree with previous studies^21,22^. While locomoting on the treadmill the alligators exhibited typical limb movements and undulations of the trunk, these resulted in the head sweeping through horizontal arcs (Fig. 2B,C).

There was a close congruence between the oscillatory movements of the alligator’s head and the pulsations in the CSF pressure (Fig. 3B–D). These locomotor-linked CSF pulsations had an amplitude (mean of 59.5 mm Hg) that was some 16 × larger than the cardiac-linked pulsations. The video records revealed no displacement of the pressure catheter or transducer during locomotion. Control trials involving manual manipulation of the anesthetized alligator or mounting the pressure transducer on remote control devices that moved over the treadmill (data not shown) all support the conclusion that these CSF pulsations are not artefactual. The close congruence between a kinematic variable (head rotation) and the CSF pulsations (Fig. 3B–D), combined with similar large amplitude CSF pulsations reported during locomotion in snakes^18^, support the conclusion that the CSF pressure pulsations reported in this study are generated by the movements of the alligators.

The physical bases for these movement-induced CSF pulsations are not clear. Crocodylians have a well-developed myodural bridge, a link between cervical skeletal muscles and the dura^23^. These suboccipital muscles would likely be contracting during head rotations, and previous experimental studies on alligators have shown that contraction of the myodural bridge alters the CSF dynamics^16^. Experimental manipulation of body cavity pressures in *Alligator* altered the CSF pressure^24^, it seems likely that changes in body cavity pressure, either ventilatory or independent, during locomotion would also influence the CSF. Additionally, the lateral undulations of the vertebral column that occur during terrestrial locomotion in crocodylians (Fig. 2A) could produce a variety of potential influences (e.g., physical displacement of the spinal cord^25^) which could alter CSF pressure.

The influence of movement/locomotion on CSF pressure has been explored in mice^26,27^ and rats^28,29^. The results of these four rodent studies have two key features in common: the onset of motion/locomotion was associated with a sharp increase in CSF pressure (to 3–4 × the resting level), and though the elevated CSF pressures recorded during motion/locomotion were quite variable, none of the published records contain anything like the sinusoidal curves reported from *Alligator* in the present study. Presumably, the absence of similar sinusoidal curves in the rodent studies reflect the substantial differences in body size and locomotor kinematics between rodents and *Alligator*. The sharp increase in CSF pressure reported at the onset of movement in rodents^26–29^ has been linked to a decoupling of cerebral arterial and venous volumes associated with increased cardiac output^30^. The CSF pressures recorded from locomoting alligators contain both sinusoidal waves, but also a gradual increase in CSF pressure (Figs. 3A, 4B). We hypothesize that the gradual increase in CSF pressure observed during movement in *Alligator* is caused by increased cardiac output leading to an increase intracranial perfusion, and that this process takes longer in *Alligator* than in mammals.

Though influenced by the unique plasticity of their cardiovascular and ventilatory systems^31,32^, the CSF dynamics in crocodylians show many similarities to what is found in humans and other mammals^22,33^. It is not our intention to argue that human movement would likely cause CSF pressure pulsations of a magnitude reported herein from alligators; the CSF dynamics in humans are more dampened and controlled^34^. At the same time, the two key results of the present study, a general increase in CSF pressure associated with movement and CSF pulsations tied to specific body movements, have both been reported in human^35^ and other animal studies^18,29^ . As such, this study argues that a complete model of CSF dynamics, whether in humans or any other vertebrate, needs to incorporate influences beyond the cardiac and ventilatory cycles.

## Materials and methods

### Live animals

Seven live sub-adult (142–165 cm total length, 8.8–14.7 kg mass) American alligators (*Alligator mississippiensis*) were obtained from the Louisiana Department of Wildlife and Fisheries. The animals were housed communally in a 29 m^2^ facility that featured three submerging ponds, natural light, and artificial lights on a 12:12 cycle. The facility was maintained at 30–33° C, warm water rain showers were provided every 20 min, which helped maintain the facility at > 75% relative humidity. The alligators were maintained on a diet of previously frozen adult rats. When the individual animals were removed from the enclosure, they were caught by noosing, then their jaws were taped using vinyl tape. The husbandry and use of the live alligators followed all applicable federal guidelines, and were approved by the IACUC of A.T. Still University (Protocol #221, approved March 2021), and are reported following the ARRIVE guidelines.

### Treadmill and treadmill training

A treadmill (270 cm long × 61 deep × 46 wide) was fabricated out of steel beams, lined with stainless steel sheeting, and fit with a custom (538 cm long × 41 cm wide) tread (Walking Belts, LLC). Each alligator was trained twice weekly to locomote along the length of the treadmill, initially with the treadmill off, subsequently with it moving in the range of experimental velocities (0.1–0.25 m/s). All training sessions were recorded using two digital video cameras (Action camera, YI Technology), one providing a dorsal view and the other a head-on view. Once all of the alligators demonstrated stable locomotor patterns on the moving treadmill, the surgical experiments were initiated. One terminal surgical experiment was performed each week; the training schedule was maintained for all of the remaining animals.

### Surgery and data collection

When the individual alligator was noosed for the surgical experiment is was induced to bite a bite pad, and the animal’s mouth was taped shut around the bite pad. Each individual alligator was placed on a stiff board (244 × 28 × 3.8 cm thick), which exceeded the maximum width and length of the alligators used for this study. Six 2.5 cm wide heavy duty straps (Northwest Tarp and Canvas) were used to secure the alligator to the board; the straps were tight enough to minimize movement of the animal but not tight enough to impede ventilation or circulation. With the alligator’s mouth held open by the bite pad, a laryngoscope was used to depress the gular valve and expose the glottis. A cuffed endotracheal tube was inserted into the larynx and connected to a custom anesthesia system that included a ventilator pump (Harvard Apparatus), Vaporstick anesthesia machine (Surgivet), isoflurane vaporizer (Surgivet), and Capnomac Ultima respiratory gas monitor (Datex-Engstrom). The alligators were maintained on a steady ventilatory pattern of 5 breaths per minute each with a tidal volume of 500 ml. Anesthesia was accomplished using 5% isoflurane. Two silver chloride surface cup electrodes (019–477,200, GRASS), coated with a layer of conducting gel (Signagel, Parker Laboratories) were placed on the lateral surface of the animal, on either side of the heart. Meloxicam (at 0.2 mg/kg) was administered into the left triceps to serve as an analgesic.

A surgical drill (MPS Powerforma, XOMED) was used to bore a 4.0 mm diameter hole through the dorsum of the alligator’s skull to expose the dura. A small incision was made in the dura to allow the passage of a pressure catheter. Surgical adhesive (Vetbond, 3 M) was used to seal the dura around the catheter, then epoxy cement was added to fill the bored hole and secure the catheter to the skull. A fluid pressure transducer (APT300, Harvard Apparatus) was rested upon, and sutured to, the large osteoderm that covers the dorsum of the alligator’s neck. The pressure transducer, and the attached pressure catheter, were filled with a reptilian Ringers solution^36^. The lead coming off of the pressure transducer was sutured along the midline of the alligator to mid-body; neither the pressure catheter, transducer, nor the transducers lead, restricted the range of head or body movements of the alligator. The pressure transducer was coupled to a strain gauge amplifier (P122, GRASS), while the EKG electrodes were connected to a DC preamplifier (P511, GRASS) The outputs from these two amplifiers were sampled at 4 kHz, simultaneously with the carbon dioxide concentration from the respiratory gas monitor, using the MiDas data acquisition system (Xcitex Inc.).

The experimental sequence for each alligator was similar. Once the alligator reached a surgical plane of anesthesia. The pressure catheter was implanted into the cranial sub-dural space and a series of baseline CSF, EKG, and (forced) ventilator cycles were recorded. With the animal still under anesthesia it was tilted 30° head-up and 30° head-down (separately), each over a 90 s duration divided into 30 s baseline, 30 s tilt, then 30 s recovery. The animal was allowed to recover from anesthesia, this is a slow (hours-long) process in alligators; the animal was actively ventilated with oxygen during recovery, but no drugs were administered to accelerate recovery. Once the animal was recovered enough to actively thrash against the restraining straps, the endotracheal tube and bite pad were removed (and the jaws again taped closed). The animal was then manually transferred to the adjacent treadmill and given time to adjust. Baseline recordings, now exclusively of CSF pressure, were taken while the animal was not moving on the treadmill. Once the alligator demonstrated coordinated voluntary movement, the treadmill was turned on and locomotor data collection commenced.

Typically 3–4 locomotor sequences were recorded from each alligator (see below). Immediately after the last locomotor sequence the treadmill was turned off and the alligator allowed to remain (stationary) on the treadmill while post-locomotor CSF pressures were recorded. The animal was then returned to the surgical board, re-anesthetized, then euthanized through cardiac excision and exsanguination. Immediately after euthanizing the animal, the pressure catheter was removed and the catheter/transducer complex calibrated.

The surgery and locomotor experiment were performed in the same small room which was maintained at > 85° F to prevent metabolic disruption to the alligators. An LED flash was located adjacent to the treadmill and provided one synchronization between the video and physiological data. A trigger switch controlled by one of the authors provided a second means of synchronizing the two data sets.

### Data analysis

The video records were analyzed using Kinovea (kinovea.org). The majority of the locomotor sequences recorded were not used for data analysis (although similar CSF patterns were evident). Sequences were excluded if the alligator failed to perform at least 3 complete footfall (step) sequences, collided with the end or sides of the treadmill, was physically contacted by either researcher, or exhibited locomotor kinematics (velocity, joint angles, etc.) that differed from those recorded during the final training sessions. Ultimately, 11 locomotor sequences were analyzed; these had a mean duration of 13.12 s (s.d. = 4.87). The physiological data were quantified with Midas. The data were exported into SpectraPlus (Pioneer Hill Software) for power spectral analysis, and were exported to EXCEL for analysis.

To compare the frequency patterns of the head oscillation and CSF pressure traces, both traces were mathematically adjusted to have a mean of 0 and equal maximum amplitude. Using the timing data from the CSF recordings, a parameter optimization procedure was performed to find the best fit between a sinusoidal model [pulsation pressure = (pulse amplitude + (pulse waveform * (sine*(time—temporal offset))))-amplitude offset] and the experimentally-recorded CSF pulsation pressure data. To compare the rates of change we used the unadjusted data records and compared the slopes of the head oscillation data (which yielded velocity of head rotation) to the slopes of the CSF pulsation curve (which yielded the rate of CSF pressure change).

## Supplementary Information

Supplementary Information 1.

Supplementary Information 2.

Supplementary Information 3.

Supplementary Information 4.

Supplementary Information 5.

Supplementary Video 1.

## Data Availability

The data analyzed for the study, beyond what was provided in the Supplementary Files, is available from corresponding author on reasonable request.
