# Supplementary figures and images for: Treadmill locomotion in the American alligator (Alligator mississippiensis) produces dynamic changes in intracranial cerebrospinal fluid pressure
Source: Sci Rep. 2022 Jul 12;12:11826. doi: 10.1038/s41598-022-15918-9 (PMC9276781; doi:10.1038/s41598-022-15918-9)

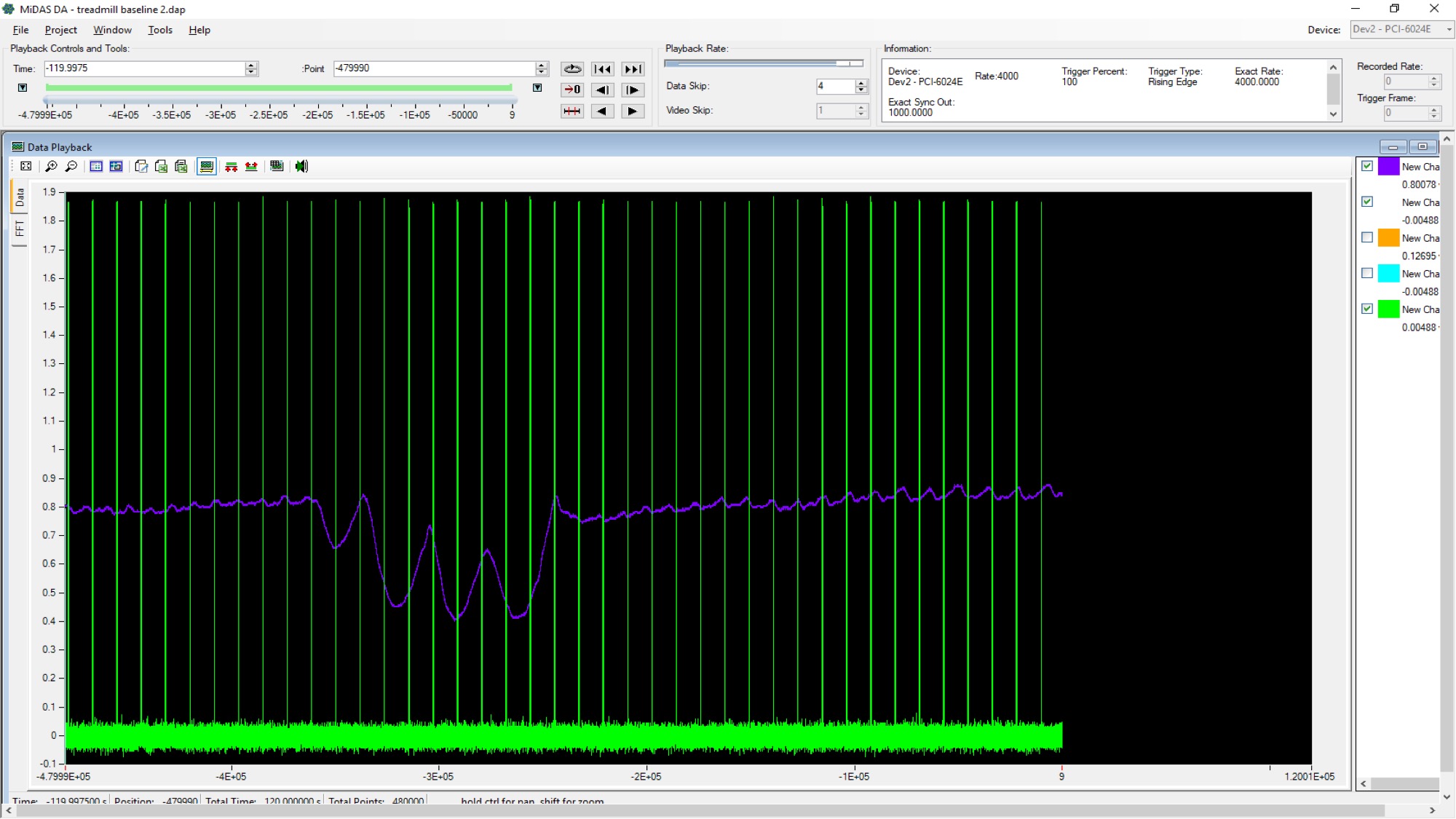

Supplement: Supplementary file 2 — Supplementary Information 2. [file 41598_2022_15918_MOESM2_ESM.jpg]

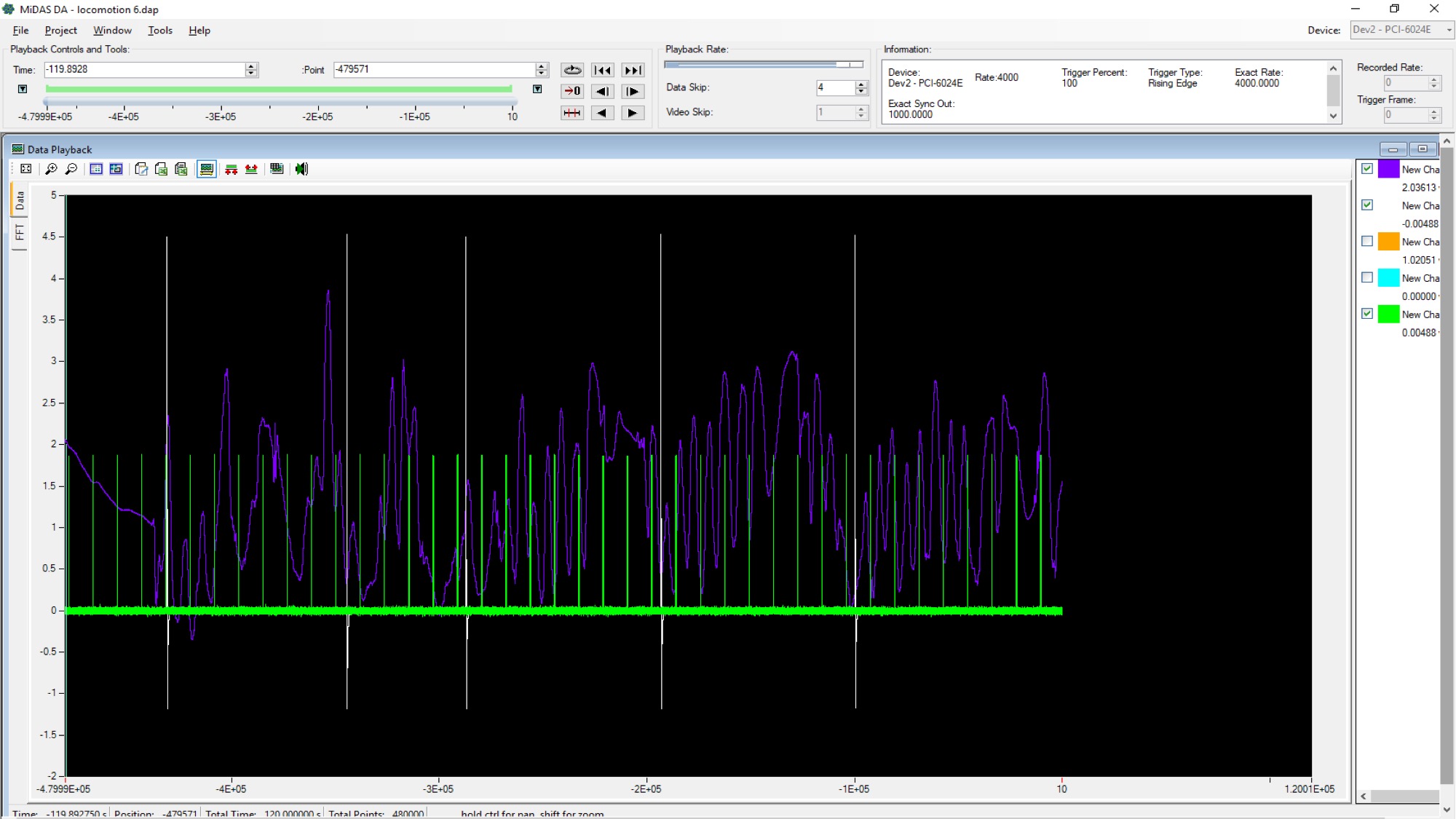

Supplement: Supplementary file 5 — Supplementary Information 5. [file 41598_2022_15918_MOESM5_ESM.jpg]
