# Supplementary material for: Treadmill locomotion in the American alligator (Alligator mississippiensis) produces dynamic changes in intracranial cerebrospinal fluid pressure
Source: Sci Rep. 2022 Jul 12;12:11826. doi: 10.1038/s41598-022-15918-9 (PMC9276781; doi:10.1038/s41598-022-15918-9)
Supplement: Supplementary file 3 — Supplementary Information 3. [file 41598_2022_15918_MOESM3_ESM.docx]

Explanation of Supplemental Material:

**Baseline Image** is a screenshot of the dataset as displayed by our data acquisition system. On this screen the CSF pulses are in blue and clearly show the cardiac-linked pulsations (note the scale of the Y-axis). These data were recorded 2 Nov. 2021 at 1:51 PM.

**Baseline Data** is an EXCEL spreadsheet of the raw data presented in the Baseline Image.

**Locomotion Image** is a screenshot of the dataset as displayed by our data acquisition system. On this screen the CSF pulses are in blue and these clearly show the locomotion-linked pulsations (note the scale of the Y-axis). These data were recorded 2 Nov. 2021 at 2:33 PM.

**Locomotion Data** is an EXCEL spreadsheet of the raw data presented in the Locomotion Image.

**Locomotion Video** is a compressed digital video of the alligator on the treadmill, this is the video sequence corresponding to the **Locomotion Data** and **Locomotion Image** files.

In the two EXCEL data files:

Column A (time) — time code in increments of 0.25 msec, total of 120 s

Column B (index) — data points, total of 480,000 (120s sampled at 4KHz)

Column C (chan. 1) — CSF pressure

Column D (chan. 2) — trigger marker. You can visualize the trigger marker being pushed near 1:58:00 in the video sequence, this corresponds to a spike in chan. 2

at -24.8818 visible as the white trace on the **Locomotion Image**.

Column E (chan. 3) — used for EKG recordings, inactive during locomotion

Column F (chan. 4) — used for exhalatory CO_2_ monitoring, inactive during locomotion

Column G (chan. 5) — sync. pulse. Evident as the green trace in the two images, and as the LED flash in the video record.
